# Remarks on Febrile Contagion

**Published:** 1789

**Authors:** James Lucas

**Affiliations:** one of the Surgeons of the General Infirmary at Leeds, and Member of the Corporation of Surgeons in London


III. *Remarks on febrile Contagion. By Mr.* James Lucas, *one of the Surgeons of the General Infirmary at Leeds, and Member of the Corporation of Surgeons in London.*

FROM a ſtrict examination of regiſters, as well as from common obſervation, it is evident that no diſeaſe whatever, the plague excepted, has proved ſo deſtructive to this iſland as the ſmall-pox: and, from an idea that infection tion diffuses itself through the air of a town or country, attempts to check contagious diseases have been not only much neglected, but even discouraged. It has been proved, however, by many satisfactory observations, that contagion is more frequently spread by contact with the patient, or infected apparel, than by a morbid state of the air; and hardly any one, perhaps, would venture to deny the importance of a plan for stopping the progress of febrile infection, although the neglect of promoting such a plan is very obvious: so true it is that the most valuable discoveries, instead of being received with gratitude, have often the prejudices of mankind, for a length of time, to combat before they become generally adopted.

There is no febrile contagion so well understood as that of the small-pox, nor any countr' where the means of preventing its fatality hav: been more successfully employed than in tls island. However sanguine it may appear I have, with my worthy and intelligent friend Dr. Haygarth, little doubt that the disorder right be so far eradicated as never to prevail except when imported from some other contry. Such power of managing one febrile in:ction might lead us, by equal caution, to checkhe ravages of other diſorders, which ſpread their baneful influence without controul. Infectious fevers, by being chiefly confined to the poorer claſs, often prevail for a length of time without exciting much alarm, or without their fatality being attended to ; but ſhould a few in higher rank receive the infection, then the diſorder is deſcribed in moſt exaggerated terms. Yet it is almoſt impoſſible to prevent ſomething being brought, or ſome perſon coming from an infected houſe, when ſuch complaints are very prevalent, ſo that the danger becomes general. Our utmoſt endeavours, therefore, ſhould be exerted to prevent the ſpreading of ſuch fatal diſeaſes, eſpecially as the contagion may often be more under management than has been generally ſuppoſed.

Unable to ſucceed in promoting meaſures ſimilar to the benevolent plan which has for ſeveral years been eſtabliſhed at Cheſter, I have contented myſelf with occaſionally practiſing Dr. Haygarth's rules ; and have alſo recommended the practice to my acquaintance, ſhould the ſmall-pox become prevalent in their neighbourhood. Knowing that one of the gentlemen allude to, who is a clergyman, had not only taken much pains to eſtabliſh ſuch a plan, but had received from it much ſatisfaction, I requeſted quefted him to tranfmit to me a narrative of his proceedings, which I fhall beg leave here to infert.

" In the month of May, 1786, I was in-
" formed by the woman who nurfed a child of
" mine, that the fmall-pox had juft begun to
" appear in a poor family not far diftant from
" her houfe. I defired the family neither to af-
" fociate with their neighbours, nor to permitany
" one who had not had the fmall-pox to come
" near them ; and I alfo called upon every fa-
" mily in the village to requeft that they would
" be equally careful not to have any communi-
" cation with the infected houfe. My direc-
" tions were as punctually followed as I could
" have expected ; but the diforder proved fatal
" to one out of two children in that family. A
" child refident under the fame roof, and a
" young girl who imprudently vifited the in-
" fected corpfe, were the only perfons who
" caught the infection, which I attribute to the
" precautions ufed.

" In October, 1787, I was informed that a
" child had been a day or two before brought
" from a diftant village extremely ill with the
" fmall-pox. By an equal attention to Dr.
" Haygarth's inftructions, every perfon efcaped
" the

" the contagion, except a little child in the

" ſame habitation, although it was a public

" houſe, and in the center of the village. In

" the beginning of December following I heard

" that the ſmall-pox was very rife in a neigh-

" bouring town, as well as in ſome adjacent

" villages; to one of which I was told a pa-

" riſhioner of mine was gone to nurſe her

" grandſon, who was dangerouſly ill with the

" ſmall-pox. I was much alarmed leſt the diſ-

" order ſhould be brought amongſt us, and

" thereby defeat a general inoculation, which I

" intended to offer the enſuing ſpring.

" Soon after I was informed that the boy was

" dead, and that his nurſe was expected home,

" where there were three children who had not

" yet had the ſmall-pox, and who were alſo in-

" tended to partake of the propoſed inocula-

" tion. I objected to any of the family con-

" veying my inſtructions to the place where the

" diſorder prevailed, although I found them

" deſirous of uſing every precaution; but I re-

" queſted that the nurſe ſhould, upon her re-

" turn, put off the cloaths ſhe might then wear,

" at a relation's houſe, where there was no per-

" ſon capable of being infected; that her linen

" ſhould be cautiouſly waſhed; and the reſt of

" her

" her apparel fumigated, and expofed to the

" air. By all thefe precautions being cheerfully

" complied with, I had the fatisfaction to find

" that no one caught the infection.

" In the month of March we were again dif-
" treffed by a neighbour's child being brought
" from a diftant fchool with a very malignant
" kind of fmall-pox, which foon proved mor-
" tal; yet, except two children in the fame
" family, every one efcaped being infected,
" although a perfon had taken his two children
" into a room where one of the patients was
" with a view for them to take the diforder.
" From the information you had given me, I
" was in hopes the patient was not fo far ad-
" vanced in the difeafe as to communicate in-
" fection, which proved to be the cafe, and
" the young folks were inoculated a few weeks
" after.

" I had no fooner taken down the names of
" fuch children in the village as offered for ino-
" culation, than I was requefted by feveral per-
" fons to extend the fame privilege throughout
" the parifh. As fuch a plan exceeded my in-
" tended limits, I acquainted a noble Lord
" with my proceedings, who immediately ap-
" proved what had been done, and, in the moft

 " polite

" polite manner, requested that he might be al-
" lowed to be at the sole expence of executing
" a scheme which every family to whom he had
" applied had, not many years before, peremp-
" torily refused.

" Notwithstanding the unfavourable appea-

" rance of some of the children, including a

" few private patients, near eighty were inocu-

" lated, without even any apparent danger;

" whilst two, out of five, who caught the na-

" tural infection died. As I had three of my

" own children inoculated by the gentleman

" who undertook the general inoculation, and

" at the same time, I became not a little anxi-

" ous that no want of success should happen

" from any failure in enforcing the necessary

" directions. Experience demonstrated the ad-

" vantage of some person exerting himself in

" admonition as well as caution. I cannot too

" strongly solicit a similar attention in the clergy

" or principal persons of either town or coun-

" try, being fully persuaded that if such a mode

" was generally adopted the most happy conse-

" quences would ensue; the lives and future

" health of the rising generation would be

" greatly preserved; the malignant effects of

" the disease would be so far moderated as to

"render it as little dreaded as other complaints;
"if even hopes might not be entertained of
"eradicating it from our iſland."

This gentleman, in whoſe relation the greateſt confidence may be placed, having no idea of a corpſe being likely to retain infection, at firſt failed to extend his admonitions ſo far; otherwiſe, in all probability, one, out of the only two at that time infected, might have eſcaped. In this caſe the corpſe remained in the ſame room as before death; therefore it is as probable the infection might have been taken from approaching ſome part of the cloaths, which are allowed to be more contagious than the body; and hence ariſes the neceſſity of uſing means to prevent future infection from what has been uſed about a patient in the ſmall-pox.

When we reflect on the circumſtance of the diſeaſe having broken out not only in the center of the village, but alſo in a houſe much frequented, the ſucceſs of his ſecond attempt is as ſatisfactory as the benevolent author of the preventive means could have wiſhed. In a former letter, from the ſame Clergyman, he told me that he met with a diſappointment at this time, as he had expected that his being able to point out the good effects of his firſt attempt, would have been a ſufficient argument with his pariſhioners to have produced a future cheerful compliance with his regulations; but finding this not to be the caſe, he had recourſe to the reminding them of the loſs that muſt be ſuſtained by parents who, at that ſeaſon of the year, ſhould be detained from the harveſt to nurſe their children in the ſmall-pox. This argument was no ſooner made uſe of than univerſally attended to by a ſatisfactory compliance. His diligence a few months after not only proves his full perſuaſion of the advantage, but alſo the power of checking contagion from being communicated, under very unpromiſing circumſtances. It alſo appears that his practice had a moſt unexpected and happy conſequence in diſpoſing perſons, hitherto inimical to an improved method, to become earneſt petitioners for its benefits. It may be remarked, that not one of the families would previouſly aſſent to a general inoculation, although afterwards ſuch a ſalutary ſcheme met with univerſal approbation. A ſtrong proof is alſo exhibited of the great utility ariſing from occaſional general inoculation, both in point of additional ſecurity, and alſo in not communicating natural infection, as no one was found to have caught the diſorder from any of the inoculated patients.

I have

I have endeavoured to eſtabliſh in this town a plan for preventing the ſpreading of the ſmall-pox, and alſo other febrile contagion; but although I obtained a promiſe of ſeveral contributions, yet I could not procure the neceſſary aſſiſtance and ſupport for carrying it into execution. When I have been called to a patient in the natural ſmall-pox, I have endeavoured to trace the introduction of the diſeaſe, and alſo to inculcate the preventive meaſures. In one inſtance I found that the diſorder had been brought by a vagrant, and had been communicated to three families in the ſame ſtreet: by calling upon ſuch families as ſeemed moſt expoſed, and uſing every exertion in my power, a total check to the progreſs of infection, as far as I could learn, was the conſequence. Could I have ſpared time, I wiſhed to have propoſed an occaſional inoculation, as the complaint was confined to one ſtreet. Although equally good effects from purſuing the preventive means, or from occaſional general inoculation, might not be experienced in populous towns as in villages, yet the eſtabliſhment at Cheſter fully proves that the following year conſtantly exceeded the preceding in rendering the plan more eaſily executed.

The reſult of two general inoculations in

Leeds

Leeds * has been, that the ſmall-pox has ſince been leſs frequent and leſs fatal; its introduction might more eaſily be traced, and the poorer claſs ſeem to have adopted a more advantageous method of treating the natural diſtemper.

The poſſibility of carrying Dr. Haygarth's rules into execution in villages requires ſo few exertions, and is evidently attended with ſuch happy conſequences, as to afford little doubt but the communication of the preceding narrative will have its due influence. There are

* One of theſe took place in the year 1781.

The number of inhabitants in Leeds, in that year, was 17,117; of whom 7475 (males and females) were under twenty years of age.

In the ſpace of ſix months, in that year, 462 { perſons had the ſmall-pox.

| | |
|---|---|
| Of theſe recovered — — — | 332 |
| ——— died — — — — | 130 |
| In the next ſix months were inoculated — | 385 |
| Of theſe recovered — — — — | 381 |
| ——— died — — — — — | 4 |

The number of thoſe who were ſtill uninfected was found, on a ſurvey, to be 700.

Two of the four, who died, evidently appeared, from the early commencement of the eruptive fever, to have received the natural infection previouſly to their being inoculated.

The general inoculation, ſo far from ſpreading the diſorder, appeared to put an immediate check to its progreſs.

bountiful bountiful perfons in every place, who liberally difpenfe affiftance to the indifpofed and needy; and thofe who are accuftomed to fuch vifits would feldom fail to gain early intelligence of the fmall-pox, and any dictates of theirs would be fcrupuloufly obferved from the beft of motives, gratitude and affection. The experience and fuccefs of villages, in ftopping the progrefs of infectious diforders, would tend to encourage focieties being formed in populous towns for the fame laudable purpofe.

In the year 1779 I addreffed a letter to the gentlemen who had the management of the poor in this town, recommending to them, in as ftrong terms as I could, a houfe of reception for fuch as might be feized with infectious fever, and require affiftance from the town. I was led to this ftep from having vifited feveral patients who laboured under a malignant fever, and from obferving that the fame contagion continued for many months; that eighty perfons died of the diforder in one year; that many who ftruggled through the difeafe died of other lingering complaints; that in two courts, or yards, forty perfons were affected with the fever; and that fome families had received ten fhillings a week from the poor affeffment; that fuch fuch a fcheme appeared likely to check the malignant tendency of the difeafe, and at the fame time to be more œconomical than fupporting fuch paupers at their own houfes, where no expence could afford comfort, or that prefervation which might be expected from a well-regulated temporary hofpital, which might at that time have been almoft entirely fupplied with furniture and attendants from the poor houfe. At the fame time I recommended, that, by way of afcertaining the expence of fupporting families afflicted with fever, the letter F fhould be placed to every fum fo difpofed of.

To enumerate the diftreffes of families afflicted with fever, that have fallen under my own notice, would far exceed the limits neceffary to be obferved in the prefent paper; but I cannot avoid obferving, that I have met with inftances where additional fufferings have been owing to the patients being inmates, and therefore fearful of applying for relief left they fhould be removed; and it appears to me that the poor laws which relate to fuch removals particularly require fome regulation.

Although the plan I recommended was, without any trial, rejected, left fome additional expence fhould be incurred, I am fully perfuaded that ſuch a houſe would be of as real uſe as an Infirmary, Diſpenſary, or Aſylum, and if enforced by law, under proper regulations, would conſiderably reduce aſſeſſments for the poor.

A Diſpenſary has, in many places, been inſtituted for the relief of patients whoſe complaints are inadmiſſible at hoſpitals; but, unleſs a houſe is annexed to it for the purpoſe of taking in patients labouring under febrile infectious diſorders, it does not anſwer well the purpoſe intended. If ſervants are ſeized with a malignant fever, they are often ſent home to a crowded habitation, where they not only endure much for want of a more proper aſylum, but alſo propagate the diſorder, and produce great diſtreſs to all around them. In this caſe it may be ſaid that the law requires a maſter to provide a cure for his hired ſervant, and therefore ſuch ſervant is not an object of charity; but, unleſs ſome other motive induces a maſter's attention to his ſervant, little conſolation, under ſuch affliction, is to be expected. It may be ſaid that few perſons would be diſpoſed to prefer ſuch a receptacle to their own home. The ſame objection has ariſen at the inſtitution of all ſuch charities; but it is well known that their good regulations and advantages have in a ſhort time conquered all ſuch vulgar caprice.

Medical men would be the moſt uſeful houſe viſitors, and would amply compenſate the difficulty of procuring the attendance of other truſtees. In ſuch towns as have an Inſpector, he would be the proper perſon to give notice of any prevailing infectious diſorder; and when the benefits of a houſe of reception were once experienced, the application from the diſtreſſed family would generally ſuperſede his diſcovery.

An exact account of the expences of fever at home and at this temporary hoſpital ſhould be preſerved for public ſatisfaction.

By publiſhing a report of the proceedings and preſent ſtate of houſes of reception, contributions would be obtained, and ſimilar plans promoted in other places. I cannot help obſerving, that no charitable inſtitution whatever ought to be managed without the truſtees being occaſionally under a legal obligation to report publicly the intention of the donors, and the ſeveral purpoſes for which the money has been expended, as well as the balance in hand. It is to a neglect of this ſort that we may attribute the abuſe and decline of many ancient charitable inſtitutions, and the commencement of new ones, whofe progrefs depends upon their public reports. When the late act of Parliament for inquiring into charities was made, it would certainly have been, in many refpects, more beneficial if it had rendered a publication of the ftate of each charity neceffary; and it is to be hoped that fuch an amendment will in future call the attention of our Legiflature.

As infectious diforders are, of all others, the moft fatal, and all ranks of people are acquainted with the names of prevailing diftempers, an efpecial accuracy in regiftering burials, of marking thofe that have died of fuch complaints, is of far greater confequence than an attention to chronic diforders.

The conftruction of a houfe of reception, as well as the requifite means of prevention and management, may be collected from a variety of authors: amongft which Dr. Lind on fever and infection; Dr. Haygarth on the prevention of the fpreading of the fmall-pox; Mr. Howard's different publications; Sir George Paul's work; and Mr. Day's account of the contagion at Maidftone; are highly worthy of notice.

As it appears clear that the fpreading of the fmall-pox is capable of being checked; that general inoculations may be very advantageoufly conducted even at the houfes of the patients, (although it muft be allowed that a houfe for the purpofe would be preferable); as well as that great diftrefs arifes from febrile contagion being left uncontrouled; and that it is no more in the power of medical practitioners than others to promote fuch benevolent fchemes; but, on the contrary, that they have often the undeferved odium of wifhing to try experiments; it is to be hoped that at leaft their demonftrating the benefits that may be expected will be fufficient to induce thofe who have leifure, and ability, to exert themfelves in promoting fuch laudable fchemes. The beft proof of the efficacy of any plan is the fuccefs with which it is attended; but this is a fubject in which "multum adhuc reftat operæ, "multumque reftabit."

*Leeds,*

July 23, 1789.

IV. *Cafe*

